# Supplementary material for: Cyclodextrin-Oligocaprolactone Derivatives—Synthesis and Advanced Structural Characterization by MALDI Mass Spectrometry
Source: Polymers (Basel). 2022 Mar 31;14(7):1436. doi: 10.3390/polym14071436 (PMC9003485; doi:10.3390/polym14071436)
Supplement: Supplementary file 1 [file polymers-14-01436-s001.zip › polymers-1645861-SM.pdf]

Cyclodextrin-oligocaprolactone derivatives – synthesis and advanced structural characterization by MALDI mass spectrometry

Cristian Peptu[1,3*], Diana-Andreea Blaj[1], Mihaela Balan-Porcarasu[1], Joanna Rydz[2,3*]

[1] "Petru Poni" Institute of Macromolecular Chemistry, Grigore Ghica Voda Alley, 41A, 700487, Iasi, Romania.

[2] Centre of Polymer and Carbon Materials, Polish Academy of Sciences, M. Curie-Skłodowska 34, 41-819 Zabrze, Poland

[3] Polish-Romanian Laboratory ADVAPOL, M. Curie-Skłodowska 34, 41-819 Zabrze, Poland and Aleea Grigore Ghica Voda, 41A, 700487 Iasi, Romania

*E-mail: cristian.peptu@icmpp.ro, jrydz@cmpw-pan.edu.pl

Contents

MS/MS spectrum of $[CDCL_5 + Na]^+$ – **Figure S1** – page S2

^{1}H NMR (DMSO-d6, 400 MHz) spectrum of a typical CDCL product (synthesis #2) with peaks integration - **Figure S2** - page S3

COSY spectrum of CDCL final product – **Figure S3** – page S4

HMBC spectrum of CDCL product – **Figure S4** – page S5

HSQC spectrum of CDCL product – **Figure S5** – page S6

DEPT135 spectrum of CDCL product – **Figure S6** – page S7

^{1}H NMR spectra for CDCL reaction kinetics – **Figure S7** – page S8

COSY spectrum for the sample collected after 30 min of reaction time – **Figure S8** – page S9

COSY spectrum for the sample collected after 6 h of reaction time – **Figure S9** – page S10

COSY spectrum for the sample collected after 11 h of reaction time – **Figure S10** – page S11

COSY spectrum for the sample collected after 11 h of reaction time – **Figure S11** – page S12

The agreement between the M_n evolutions determined ^{1}H NMR and MALDI MS (#2) – **Figure S12** – page S13

MS/MS spectrum of $[CDCL_5\text{-}F_1 + Na]^+$ – **Figure S13** – page S13

MS/MS spectrum of [CDCL5-F2 + Na]+ – **Figure S14** – page S14

Dispersity index evolution (#4–6) – **Figure S15** – page S15

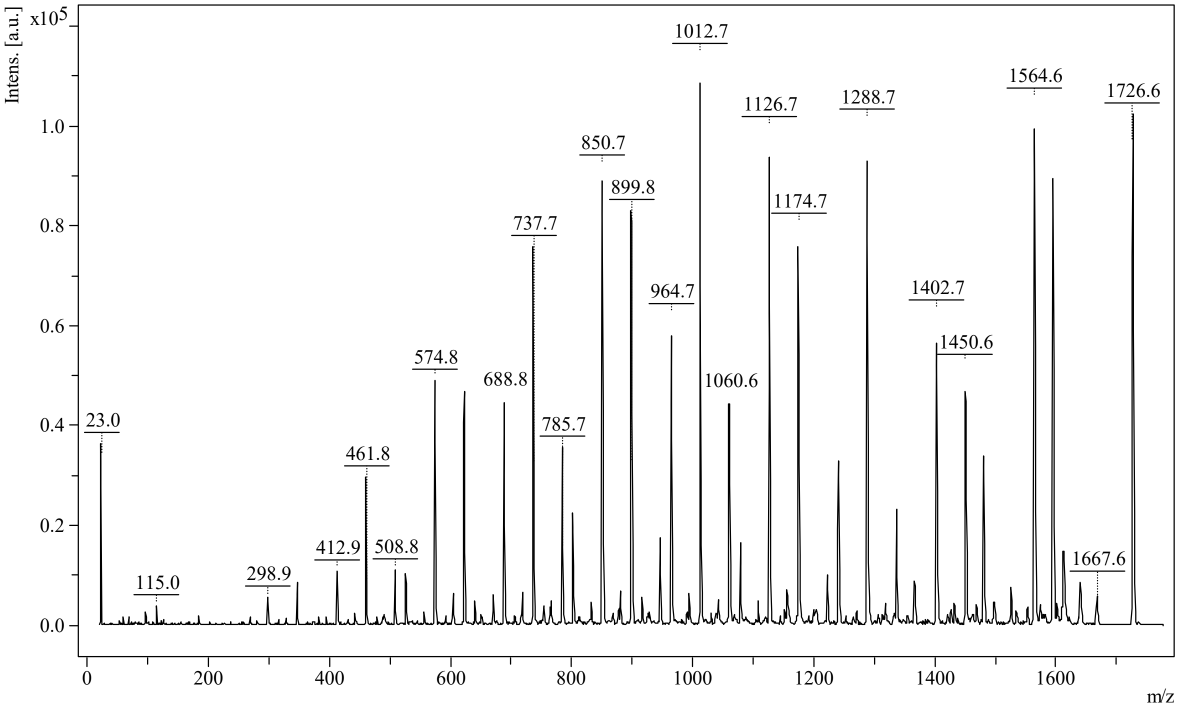

Figure S1. MS/MS spectrum of [$CDCL_5$ + Na]$^+$ (all peaks)

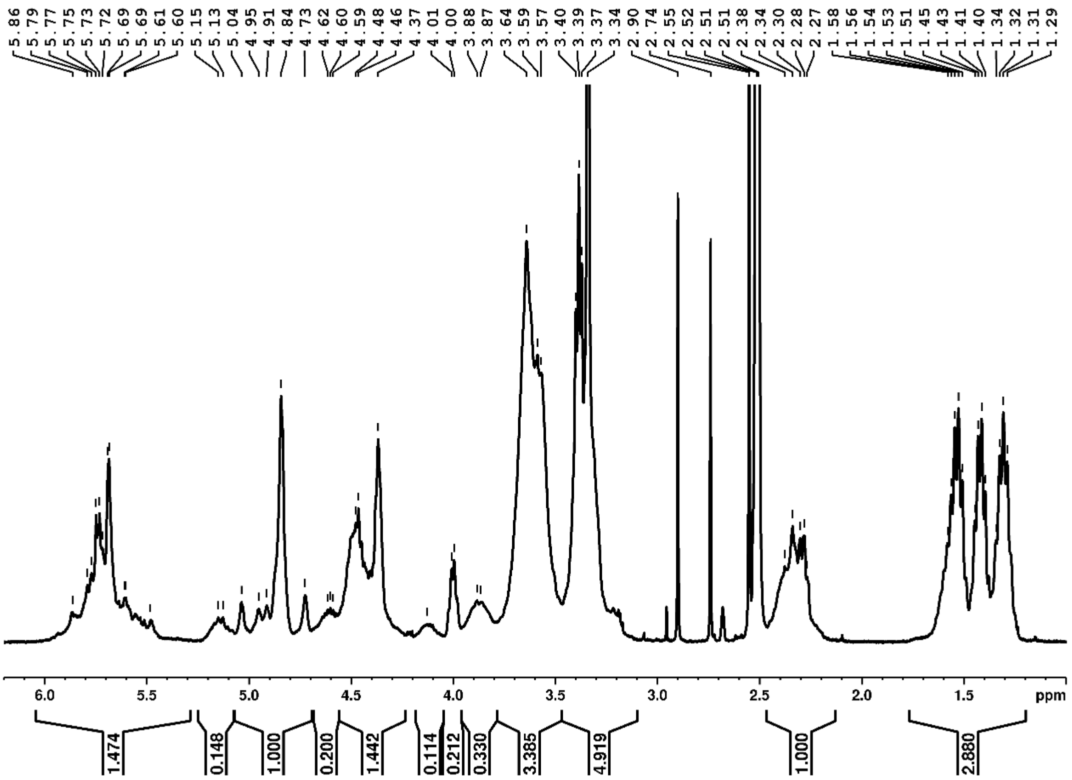

Figure S2. ^{1}H NMR (DMSO-d6, 400 MHz) spectrum of a typical CDCL product (synthesis #2) with peaks integration.

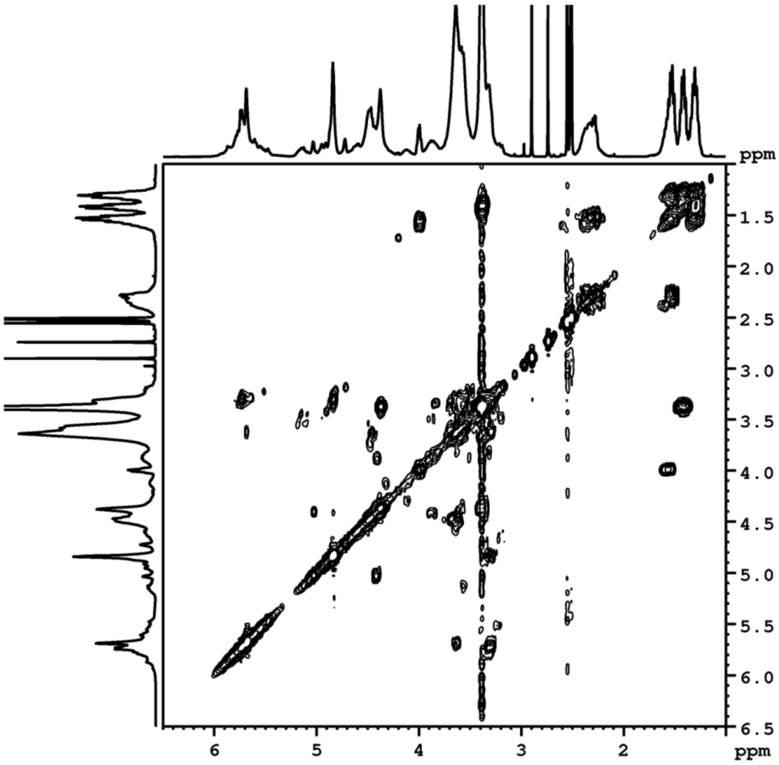

Figure S3. COSY spectrum of CDCL final product

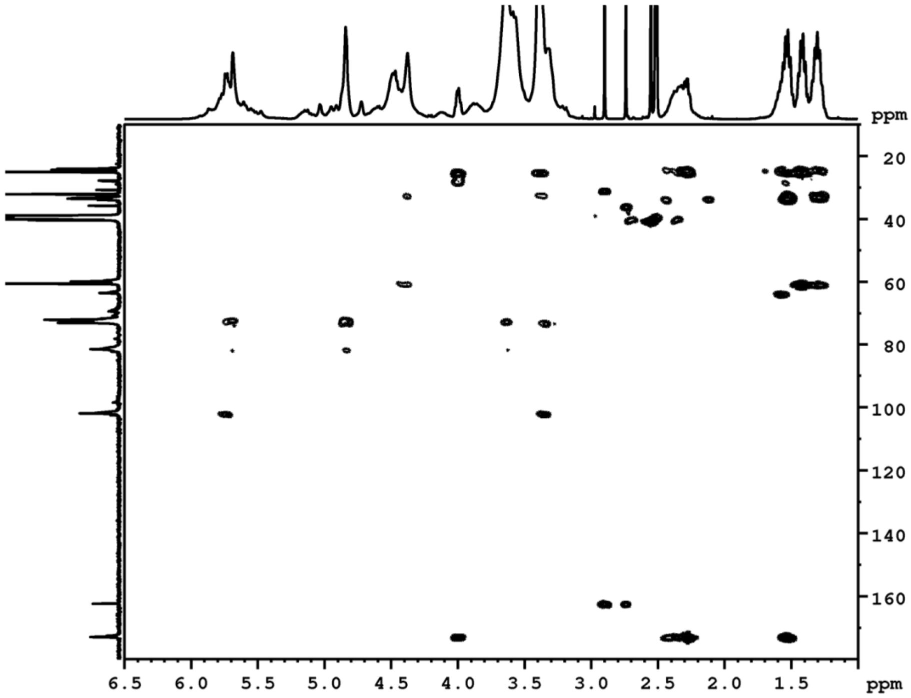

Figure S4. HMBC spectrum of CDCL product

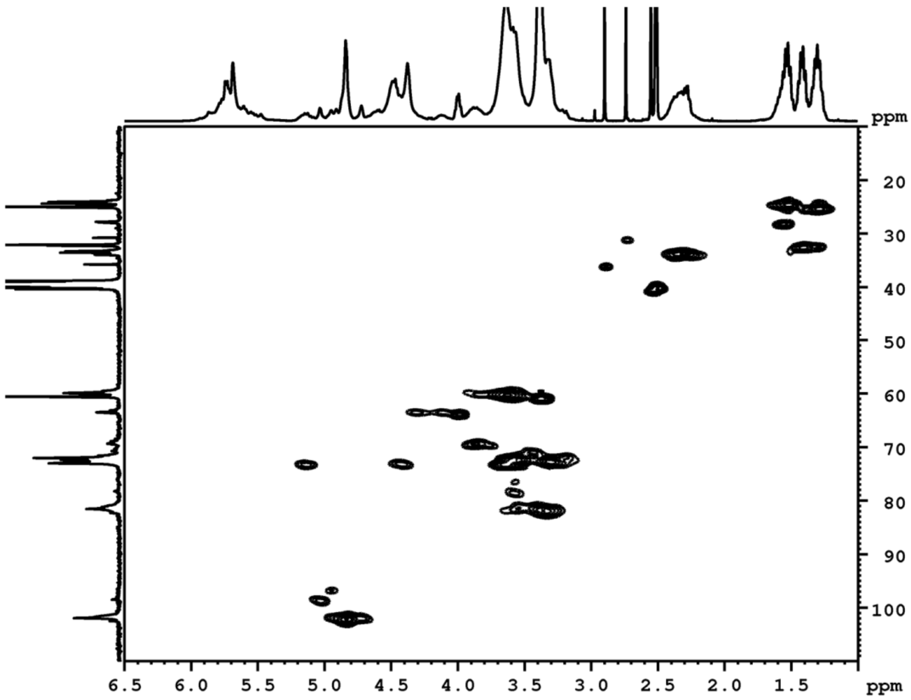

Figure S5. HSQC spectrum of CDCL product

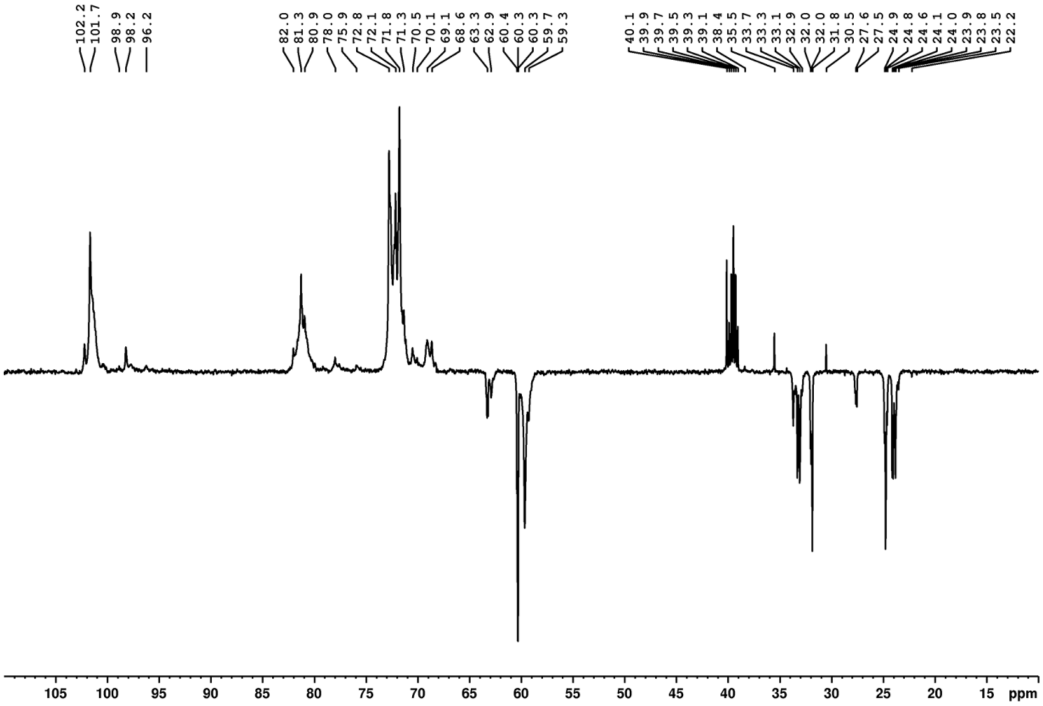

Figure S6. DEPT135 spectrum of CDCL product

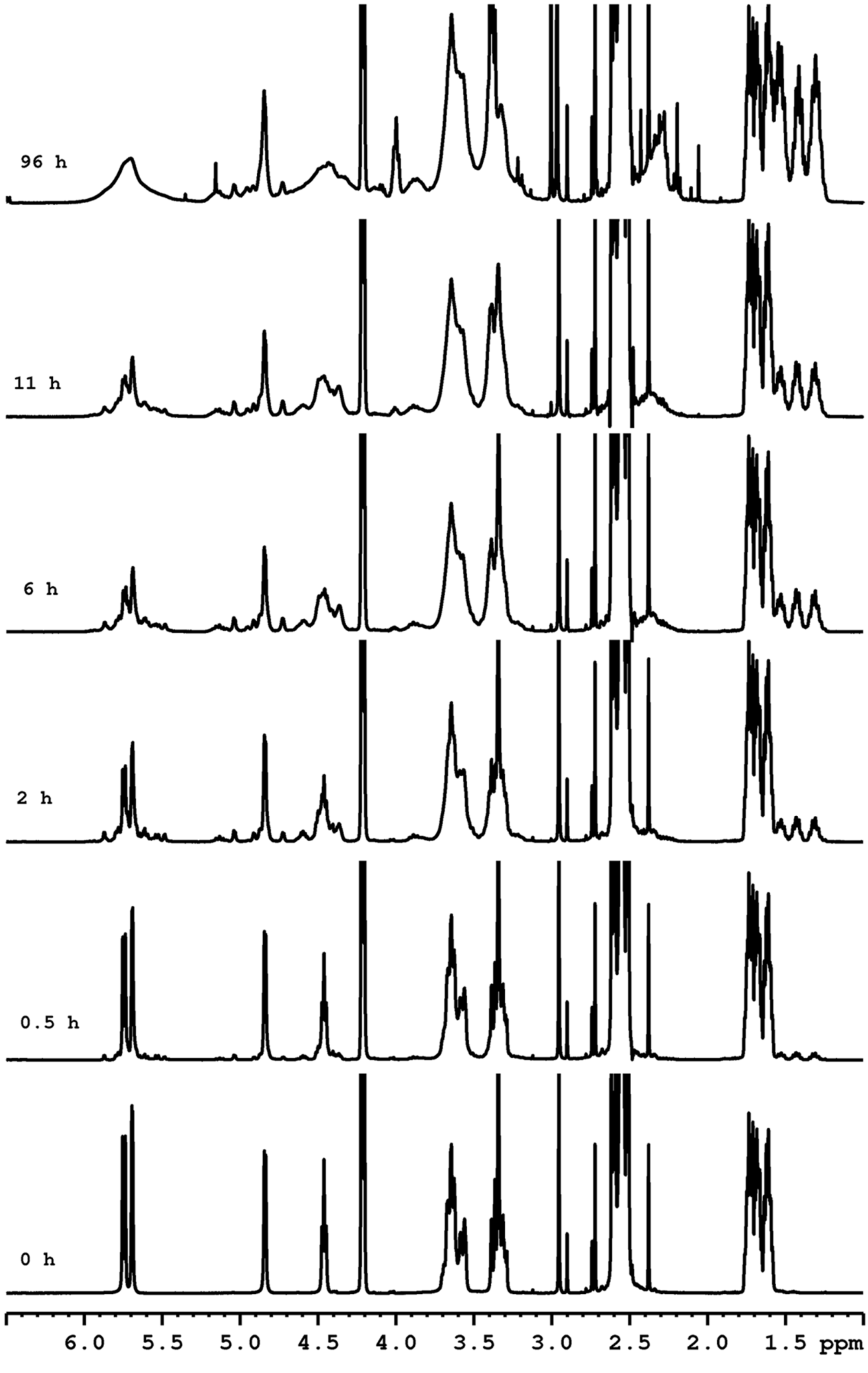

Figure S7. ^{1}H NMR spectra for CDCL reaction kinetics

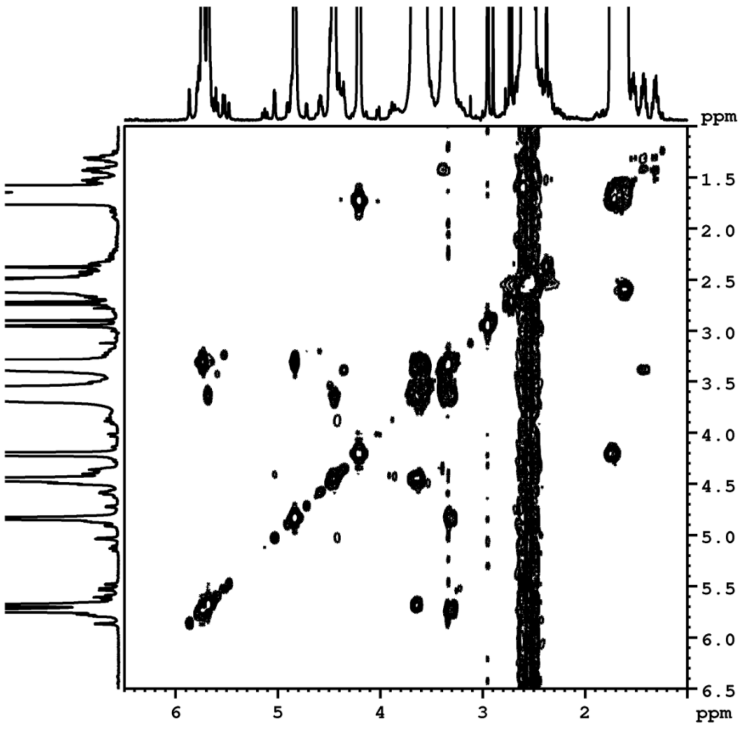

Figure S8. COSY spectrum for the sample collected after 30 min of reaction time

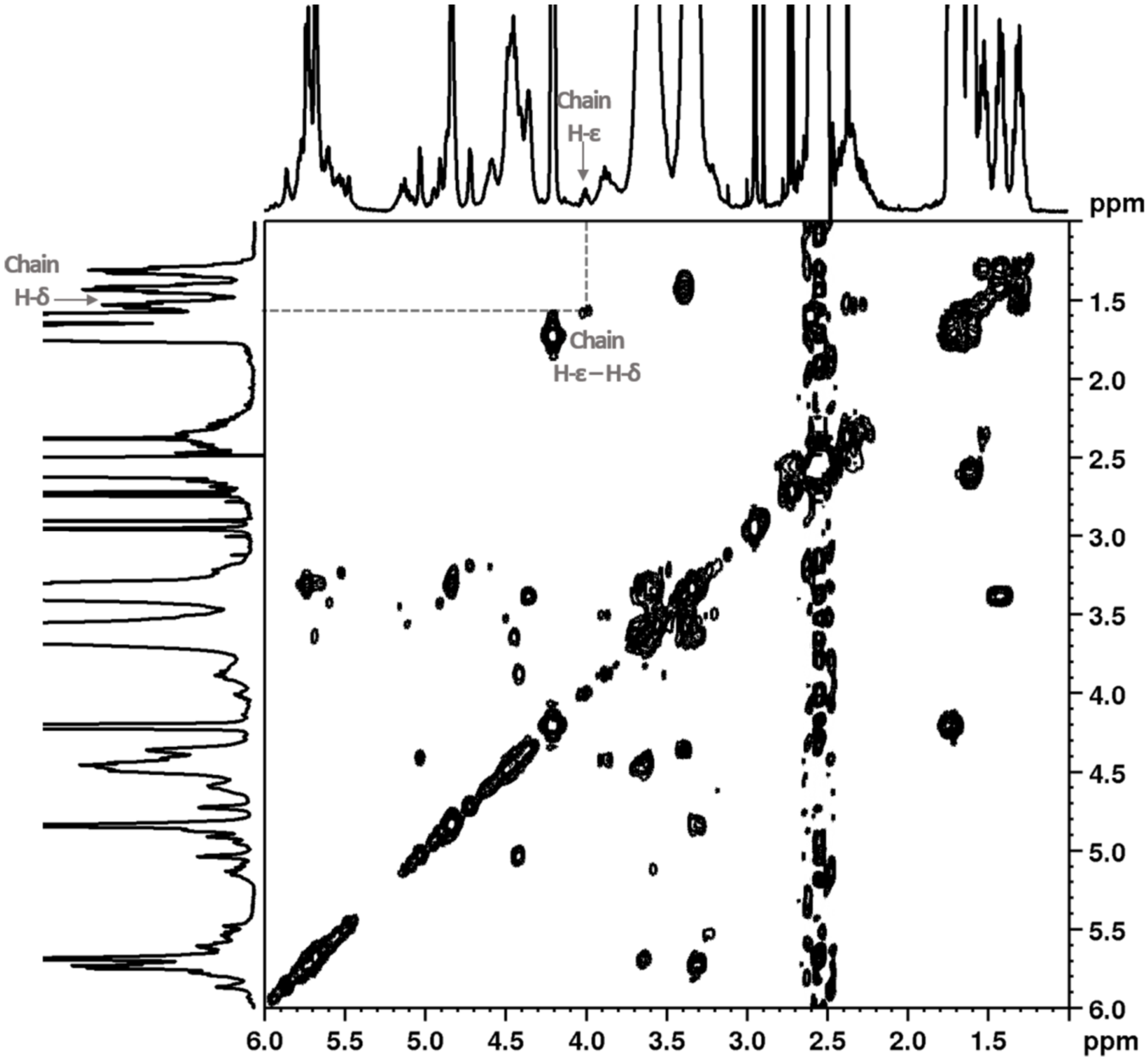

Figure S9. COSY spectrum for the sample collected after 6 h of reaction time

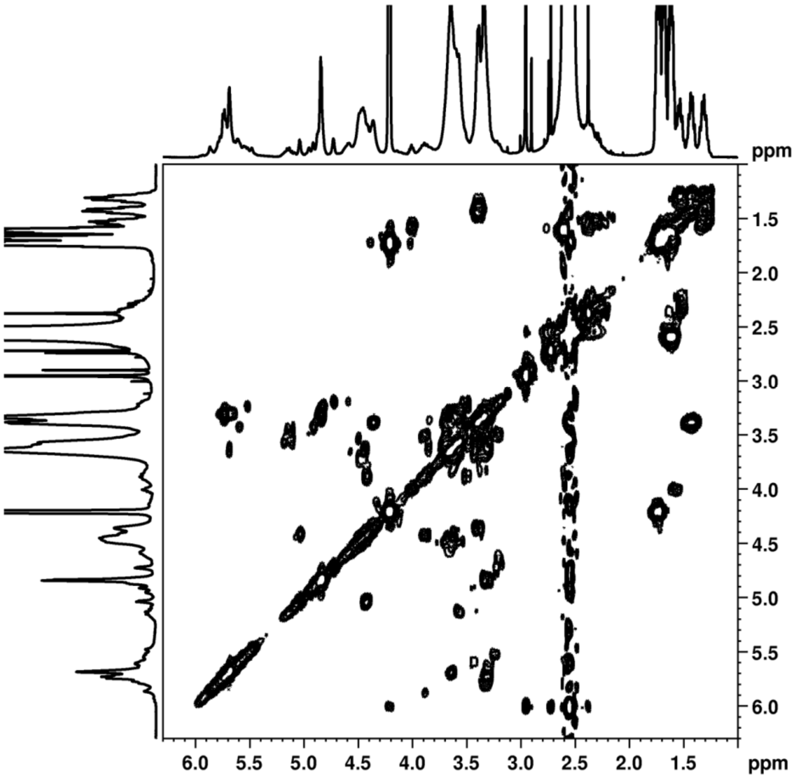

Figure S10. COSY spectrum for the sample collected after 11 h of reaction time

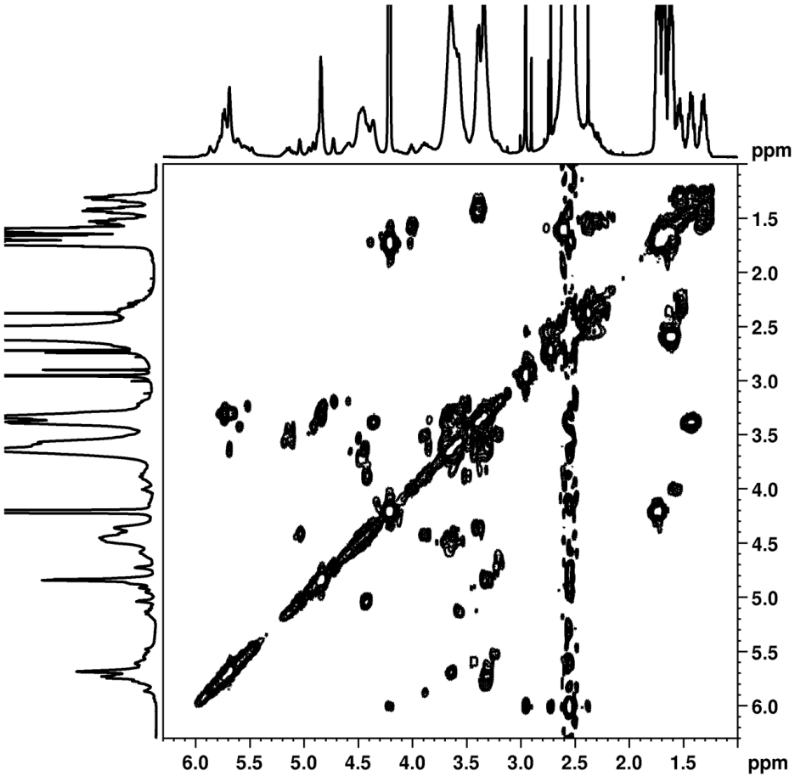

Figure S11. HSQC spectrum for the sample collected after 11 h of reaction time

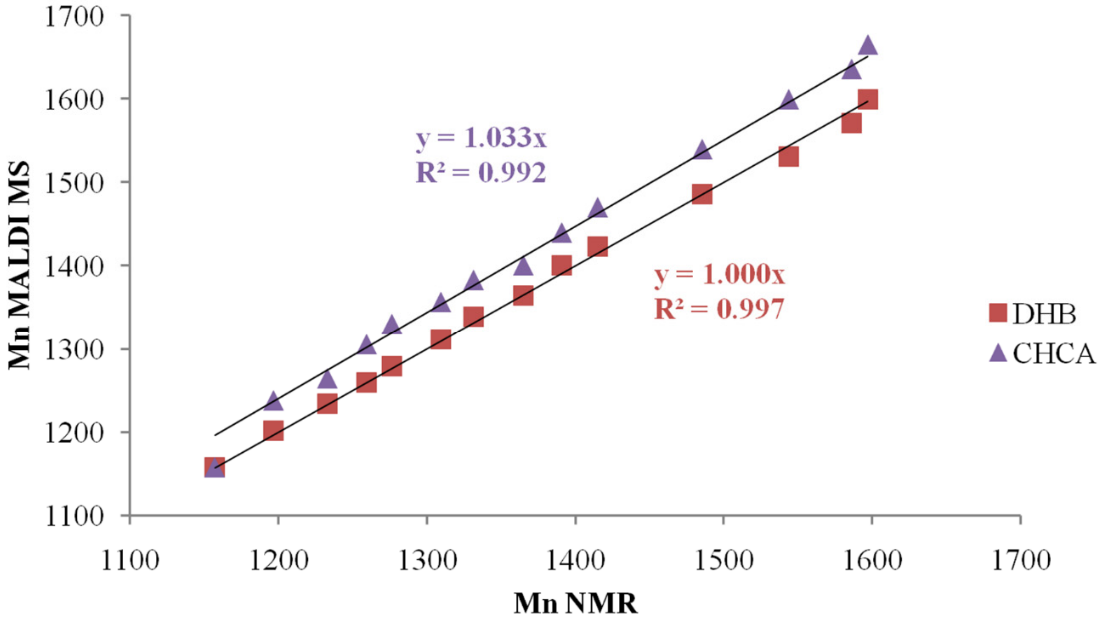

Figure S12. The agreement between the M_n evolutions determined ^{1}H NMR and MALDI MS (#2)

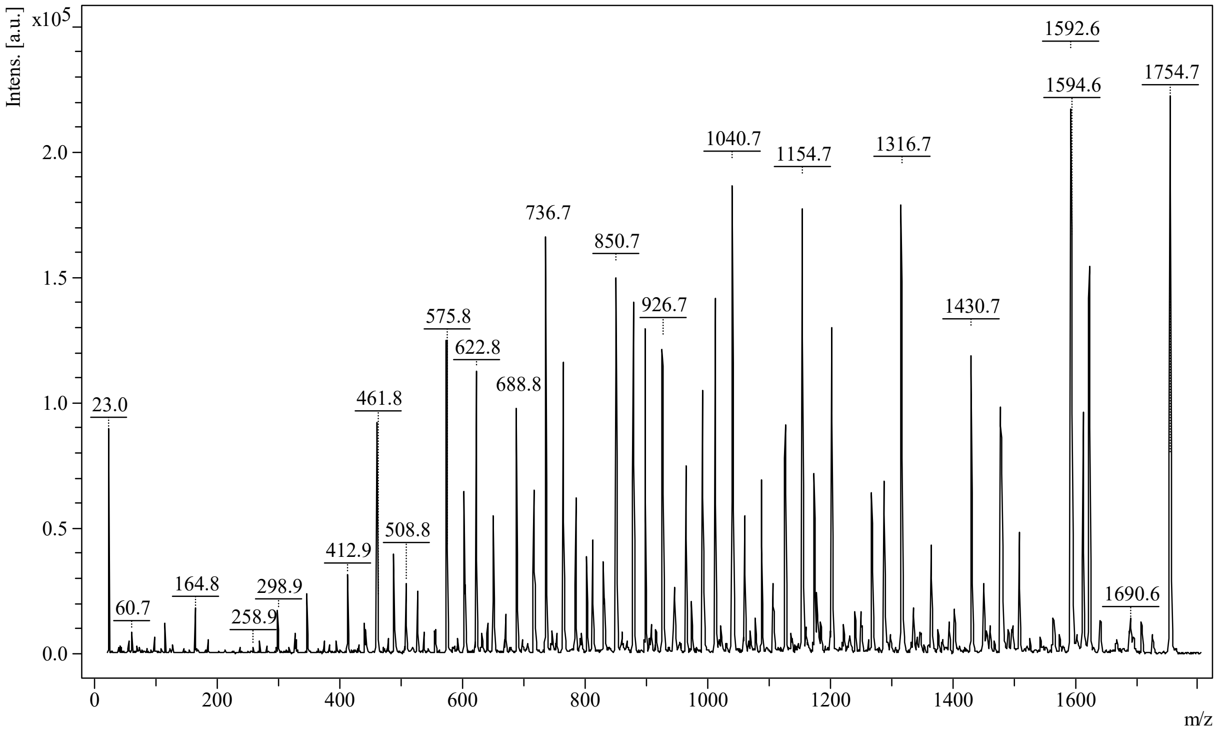

Figure S13. MS/MS spectrum of [CDCL$_5$-F$_1$ + Na]$^+$

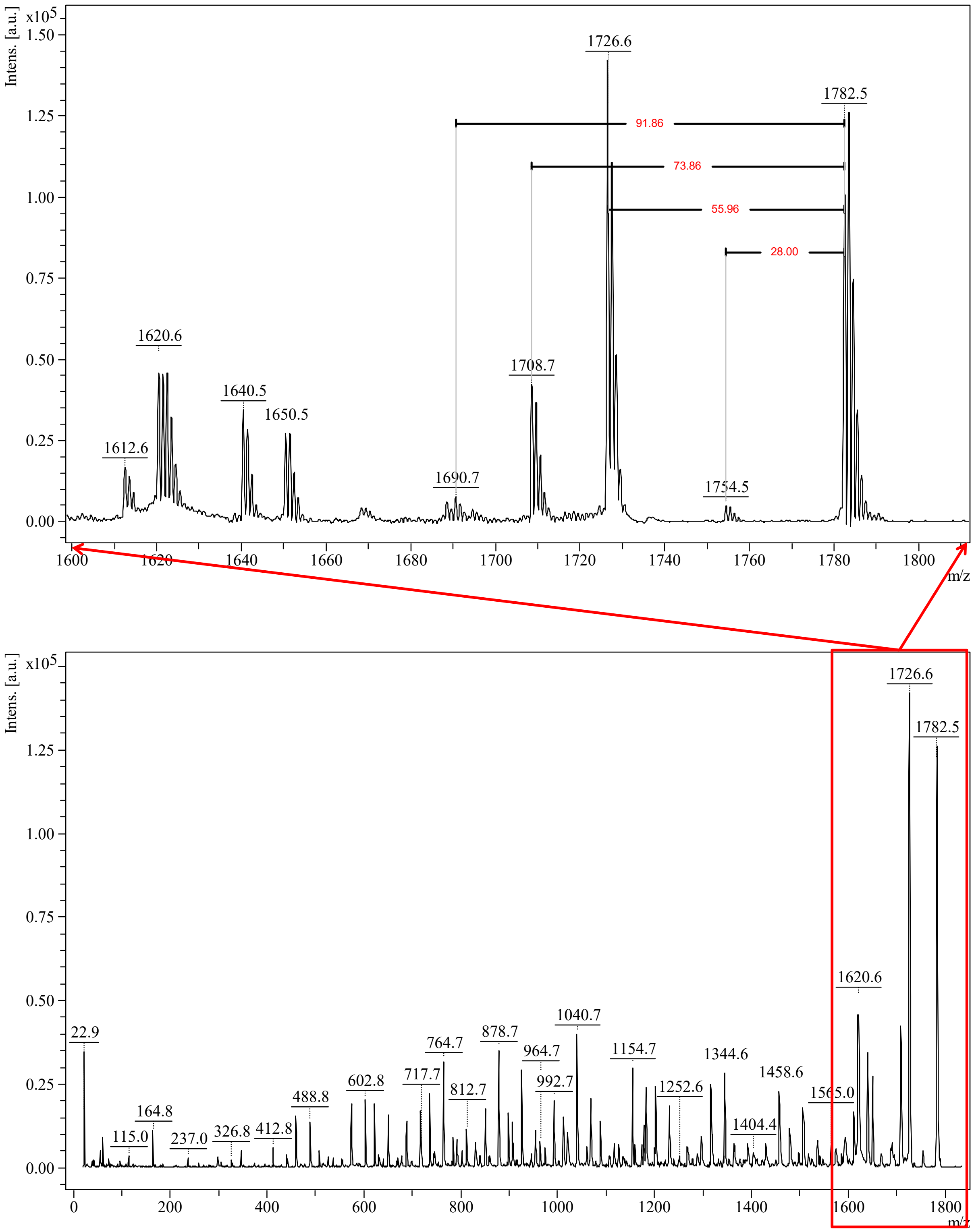

Figure S14. MS/MS spectrum of $[CDCL_5\text{-}F_2 + Na]^+$

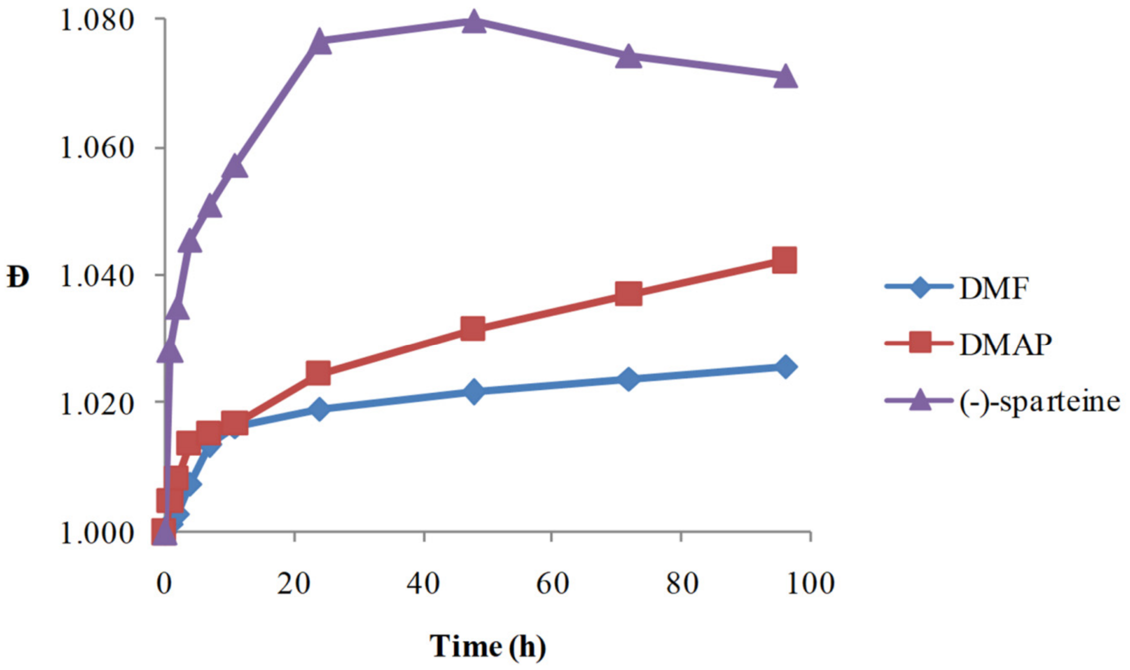

Figure S15. Dispersity index evolution (#4–6)
